# Clinical and In Vitro Safety of *Heyndrickxia coagulans* AO 1167B: A Double-Blind, Placebo-Controlled Trial

**DOI:** 10.3390/microorganisms12122584

**Published:** 2024-12-13

**Authors:** Gissel García, Josanne Soto, Antonio Díaz, Jesús Barreto, Carmen Soto, Ana Beatriz Pérez, Suselys Boffill, Ángela Gutiérrez, Raúl de Jesús Cano

**Affiliations:** 1 Pathology Department, Clinical Hospital “Hermanos Ameijeiras”, Calle San Lázaro No 701, Esq. a Belascoaín, La Habana 10400, Cuba; gisselgarcia2805@gmail.com; 2 Clinical Laboratory Department, Clinical Hospital “Hermanos Ameijeiras”, Calle San Lázaro No 701, Esq. a Belascoaín, La Habana 10400, Cuba; josanne.soto@infomed.sld.cu; 3 Statistical Department, Clinical Hospital “Hermanos Ameijeiras”, Calle San Lázaro No 701, Esq. a Belascoaín, La Habana 10400, Cuba; antoniodm@infomed.sld.cu (A.D.); angela.gtrrez@infomed.sld.cu (Á.G.); 4 Nutrition Department, Clinical Hospital “Hermanos Ameijeiras”, Calle San Lázaro No 701, Esq. a Belascoaín, La Habana 10400, Cuba; barreto.penie@gmail.com (J.B.); susubc@gmail.com (S.B.); 5 Biochemistry Department, Biology Faculty, Havana University Cuba, Calle 25 Esquina J Vedado, La Habana 10200, Cuba; carmensoto@fbio.uh.cu; 6 Cellular Immunology Laboratory, Virology Department, Tropical Medicine Institute “Pedro Kourí”, Autopista Novia del Medio Día Km 6 ½, La Habana 11400, Cuba; anab@ipk.sld.cu; 7 Biological Sciences Department, California Polytechnic State University, San Luis Obispo, CA 93407, USA

**Keywords:** safety, probiotic, humans, randomized, Heyndrickxia coagulans, clinical trial

## Abstract

(1) Background: *Heyndrickxia coagulans*, a lactic acid-producing bacterium, displays characteristics of both *Lactobacillus* and *Bacillus* genera. Clinical evidence suggests its potential health benefits. This study evaluated the safety of *H. coagulans* AO1167B as a candidate probiotic supplement. (2) Methods: Strain identification was confirmed through morphological, cultural, and genomic analyses, including 16S RNA and whole genome sequencing to assess antimicrobial resistance and virulence factors. Phenotypic tests, such as disk diffusion for antimicrobial resistance, and safety assays for cytotoxicity and hemolytic activity, were conducted. In a phase I, double-blind, placebo-controlled clinical trial, healthy adults were randomized into *H. coagulans* AO1167B and placebo groups for 60 days. Daily capsule consumption was monitored through clinical and hematological evaluations, adverse event tracking, and health surveys. (3) Results: The genome of *H. coagulans* AO1167B revealed no concerning features. Disk diffusion tests showed no antimicrobial resistance. The strain exhibited no cytotoxic or hemolytic activity, indicating in vitro safety. No significant differences in clinical or hematological parameters were observed between groups. The most common adverse event, gas, diminished over time. (4) Conclusions: *H. coagulans* AO1167B demonstrates a suitable safety profile, genetic stability, and probiotic potential for gastrointestinal health, justifying further clinical research.

## 1. Introduction

*H. coagulans* is a lactic acid–forming bacterial species first isolated and described in 1915 by B.W. Hammer at the Iowa Agricultural Experiment Station as a cause of an outbreak of coagulation in evaporated milk packed by an Iowa condensary [1]. Separately isolated in 1935 and described as *Lactobacillus sporogenes* in the fifth edition of Bergey’s Manual of Systematic Bacteriology, it exhibits characteristics typical of both genera *Lactobacillus* and *Bacillus*; its taxonomic position between the families Lactobacillaceae and Bacillaceae was often debated. However, in the seventh edition of Bergey’s Manual, it was finally transferred to the genus *Bacillus*.

The reclassification of *Bacillus coagulans* to *Weizmannia coagulans* and subsequently to *H. coagulans* reflects significant advancements in bacterial taxonomy, primarily driven by genomic and phylogenetic analyses. In 2020, a major taxonomic revision led to the establishment of the genus *Weizmannia*, named in honor of Chaim Weizmann [2]. This revision was based on genomic studies that revealed *Bacillus coagulans* had significant genetic differences from the core *Bacillus* species, particularly in traits related to spore formation and metabolic processes.

Further taxonomic refinement came in 2022, when whole-genome phylogenetic analyses provided deeper insights into the evolutionary history of *W. coagulans*. These studies demonstrated that it was more closely related to species in the newly proposed genus *Heyndrickxia*, named after microbiologist Marc Heyndrickx. Based on this evidence, *Weizmannia coagulans* was reclassified again as *H. coagulans*. This latest classification is based on phylogenetic and metabolic similarities that align *Heyndrickxia* species more closely than with those in *Weizmannia*, making the change more biologically meaningful [3].

*H. coagulans* is a Gram-positive, catalase-positive, spore-forming, motile, facultative anaerobe rod that measures approximately 0.9 μm by 3.0 μm to 5.0 μm. It may appear Gram negative when entering the stationary phase of growth. The optimum temperature for growth is 50 °C (122 °F); the range of temperatures tolerated is 30–55 °C (86–131 °F). *H. coagulans* has been added by the EFSA to their Qualified Presumption of Safety list [4] and has been approved for veterinary purposes as GRAS by the U.S. Food and Drug Administration’s Centre for Veterinary Medicine, as well as by the European Union, and is listed by AAFCO for use as a direct-fed microbial in livestock production. It is often used in veterinary applications, especially as a probiotic in pigs, cattle, poultry, and shrimp.

Numerous studies have documented the use of *H. coagulans* in humans, particularly for enhancing vaginal flora [5,6], alleviating abdominal pain and bloating in patients with irritable bowel syndrome [7,8], and boosting immune response to viral challenges [9]. Animal research provides substantial evidence supporting the efficacy of *H. coagulans* in treating and preventing the recurrence of *Clostridium difficile*-associated diarrhea [10]. Additionally, one study demonstrated that this bacterium could modulate inflammatory processes in the context of multiple sclerosis [10]. Furthermore, a specific strain of *H. coagulans* has been evaluated for safety as a food ingredient [11].

The spores of *H. coagulans* are activated in the acidic environment of the stomach, subsequently germinating and proliferating in the intestine. In several countries, spore-forming strains of *H. coagulans* are utilized as probiotics for patients undergoing antibiotic treatment [11]. Recent taxonomic revisions have reclassified this species, originally named *Bacillus* and later *Weizmannia*, into the genus *Heyndrickxia* [3].

Sunhare et al. [12] evaluated *B. coagulans* VHBAX-04, isolated from human feces, highlighting its strong tolerance to acidic environments, gastric juices, and bile salts, along with its adhesion to intestinal mucosa—traits essential for probiotic efficacy. Safety assessments confirmed the absence of toxin-related genes, and its antibiotic resistance profile indicated compatibility with antibiotic use. Genomic analysis revealed genes for fermentation, stress response, and bacteriocin production, establishing its probiotic potential.

Similarly, genome mining of *H. coagulans* BCP92 identified key genes for fermentation, stress response, and adhesion, alongside encoding a novel circular bacteriocin with antimicrobial activity. The strain lacks pathogenicity-related genes, confirming its safety and suitability for human use potential [13,14].

*B. coagulans* JBI-YZ6.3 further demonstrated robust tolerance to gastrointestinal conditions and storage stability. Genome analysis showed no toxin-related genes, and in vitro tests confirmed its non-cytotoxicity, reinforcing its potential as a safe probiotic candidate for therapeutic applications in humans and animals [15].

*H. coagulans* has been extensively utilized in veterinary applications, supported by its inclusion in the European Food Safety Authority’s (EFSA) Qualified Presumption of Safety (QPS) list [16] and its designation as Generally Recognized as Safe (GRAS) by the U.S. Food and Drug Administration’s (FDA) Center for Veterinary Medicine [17].

Additionally, the Association of American Feed Control Officials (AAFCO) lists *H. coagulans* as a direct-fed microbial, facilitating its widespread use in livestock production, particularly for pigs, cattle, poultry, and shrimp [18].

Despite its established safety in these contexts, its limited use in human food historically stemmed from two primary factors: (1) regulatory hurdles necessitating specific human-related safety data for approval in human food, and (2) a predominant focus on its application within the animal feed industry, where the demand for probiotics was significantly higher. Recent advancements in probiotic and microbiome research, coupled with growing consumer interest in functional foods, have prompted investigations into the potential of *H. coagulans* as a probiotic for human consumption. This study aims to address these gaps by evaluating the safety and suitability of *H. coagulans* AO1167B for human applications.

## 2. Materials and Methods

### 2.1. Provenance of H. coagulans AO1167B

The strain *H. coagulans* AO1167B has a well-documented provenance that underscores its historical and biotechnological significance. The parent strain was deposited in 1950 by N. R. Smith at the Agricultural Research Service (ARS) of the USDA in Beltsville, MD, USA, under the identifier B-1167. The strain was part of a collection curated for its agricultural and microbial research value.

AO1167B represents a subclone derived from this original deposit. It was selected through a screening process aimed at identifying strains with desirable traits for industrial and biotechnological applications. These included resistance to digestive system conditions such as lysozyme, gastric acidity, and bile salts, making it a suitable candidate for probiotic applications.

The strain is currently preserved in the ARS Culture Collection (NRRL), Peoria, IL, USA, where it is cataloged under the designation B-68301. Its documented lineage and selection process provide assurance of its purity and genetic integrity, highlighting its suitability for use in modern research and commercial applications.

### 2.2. Culture Conditions of Bacterial Strain

A lyophilized stock culture of H. coagulans AO1167B was suspended in TGY broth under aerobic conditions for 24 h and streaked onto TGY agar to check for purity. Single colonies were used to prepare a seed inoculum for large-scale production. The seed culture was diluted 1:10 into a larger volume of TGY and incubated for 3–5 days. The bacterial cell mass was then concentrated by centrifugation at 6000× *g*, lyophilized, and milled into a fine powder. The bulk powder was quantified for spore count using flow cytometry by BioForm Solutions, Inc. (San Diego, CA, USA). The bulk powder was stored in sealed mylar bags at −20 °C and encapsulated at 2.5 ± 0.1 billion AFU per capsule.

### 2.3. Phenotypic Characterization of the Organism

To evaluate tolerance to simulated gastric juice, overnight bacterial cultures were harvested, washed, and suspended in a 2× SES (Simulated Gastric Solution) containing pepsin. The simulated gastric juice was prepared by dissolving pepsin (3 g/L) in saline solution (0.5%, *v*/*v*) and adjusting the pH to a range of 1.8 to 5.0 using 12N HCl. The solutions were sterilized by filtration through a 0.45 μm membrane filter (Gelman Science, Ann Arbor, MI, USA) [19]. To replicate gastric conditions, the pH was gradually lowered from 5.0 to 1.8 using HCl, and bacterial cultures were incubated at each pH level for specific time intervals. Viable cell counts were measured at 0, 20, 40, 60, and 90 min to assess survival over time. All experiments were performed in triplicate [19].

Bile resistance was evaluated by growing the bacteria in broth containing various concentrations of bile (0.06% to 1%). After 24 h of incubation at 37 °C, the bacterial growth was measured by optical density (OD600) and compared to a control without bile. Results were expressed as a percentage of growth relative to the control, and all assays were performed in triplicate [20].

Antimicrobial susceptibility testing (AST) was conducted, and results interpreted in accordance with the guidelines provided by the CLSI M100, 33rd edition for the Disk Diffusion Method [21]. The antimicrobials used to assess the AST, including Ampicillin, Chloramphenicol, Clindamycin, Erythromycin, Gentamicin, Kanamycin, Streptomycin, Tetracycline, and Vancomycin.

### 2.4. Genotypic Characterization of the Organism

Hybrid sequencing was conducted at EzBiome (Gaithersburg, MD, USA). Briefly, genomic DNA were extracted, quantified using the fluorescence-based Qubit dsDNA DNA Quantification System (Thermo Fisher, Waltham, MA, USA), and sequenced using both an Illumina NextSeq 2000 platform (2 × 150 bp paired-end reads) and a Nanopore PromethION R10.4.1 flow cell (Eugene, OR, USA). For Illumina sequencing, libraries were prepared using the NEBNext^®^ Ultra™ II FS DNA Library Prep Kit (Illumina, San Diego, CA, USA). Nanopore sequencing libraries were generated using v14 library preparation chemistry without fragmentation or size selection.

Illumina reads were trimmed to remove adapters using BBDuk from BBtools v39.06 (BBMap project), followed by quality trimming and filtering with fastp v0.21.1 [22]. Nanopore reads were filtered using Filtlong v0.2.1 (--min_length 1000 --keep percent 95) to remove the 5% lowest-quality reads (Filtlong GitHub). Assembly of Nanopore reads was performed using Flye v2.9.2 [23]. Post-assembly polishing of the Nanopore assembly was conducted with Pilon v1.23 [24] using the clean Illumina reads under default parameters. 

The relationship of *H. coagulans* AO1167B to extant probiotic strains was assessed through whole-genome phylogenetic analysis. This was accomplished using the BLAST+ (Basic Local Alignment Search Tool) platform v2.15.0, which enabled the comparison of the AO1167B genome with a database of known genomes [25]. By aligning and evaluating sequence similarities, a phylogenetic tree was constructed to elucidate the evolutionary relationships between AO1167B and other strains. This approach allowed for the identification of the closest genetic relatives and provided insight into the taxonomic positioning of AO1167B within the broader context of probiotic species.

The assembled genome of AO1167B was utilized as a query in a BLASTN [25] search to identify the most closely related strains based on nucleotide sequence similarity. This search yielded a set of aligned sequences from homologous strains, which were then used to construct a phylogenetic tree using the Neighbor Joining model [26]. Strains that clustered within the same branch of the tree as AO1167B were its closest evolutionary relatives, suggesting shared ancestry and similar genomic characteristics. PlasmidFinder was used to detect the presence of plasmids in the assembled genome [27].

To identify genes closely matching previously documented antibiotic resistance genes, the assembled genomes of *H. coagulans* AO1167B were analyzed against the CARD database [28]. This curated database includes acquired antibiotic resistance genes reported in scientific literature, encompassing Gram-positive and Gram-negative bacteria, including pathogenic species.

The assembled genome of *H. coagulans AO1167B* was also evaluated for the presence of known virulence factors, using the Virulence Factor Database (VFDB) [29]. A total of 32,670 sequences were searched.

To explore the existence of potential biogenic amine-producing proteins, the annotated genome of *H. coagulans* AO1167B was examined for genes (Table 1) known to be linked with adverse events when present in food [30].

### 2.5. Hemolytic Activity

Hemolytic activity was measured by hemoglobin release (HR) as described by Lefevre et al. [31]. Briefly, human blood obtained from healthy volunteers was centrifuged (3000× *g* for 5 min) to isolate the erythrocytes. The suspension of fresh erythrocytes obtained was washed and resuspended in 1 mL of buffered phosphate solution (PBS). The concentration of the suspension was adjusted by adding PBS to obtain an absorbance of 0.7 at 490 nm, obtained by adding 1 mL of the standard to 14 mL of distilled water. For the hemolysis test, different concentrations of the compound to be tested were added to the erythrocytes. After 1 h of incubation at room temperature, the samples were centrifuged, and the supernatant was added to the wells of a 96-well polystyrene plate. The supernatant of erythrocytes in PBS1X was used as a negative control, and the supernatant of erythrocytes in distillate water was employed as a positive control.

For the hemolysis assay, varying concentrations of the test substance were added to a suspension of erythrocytes. After a 1-h incubation at room temperature, the samples were centrifuged, and the supernatant was carefully transferred to a 96-well plate. Phosphate-buffered saline (PBS) was used as a negative control, while distilled water served as a positive control, inducing complete hemolysis. The release of hemoglobin from erythrocytes was measured by recording the absorbance at 490 nm using an automated absorbance microplate reader (BioTek ELx800, Thomas Scientific. Swedesboro, NJ, USA). The percentage of hemolysis was calculated using the following formula:

$$HR\%=\left(\frac{\left(OD\;sample-OD\;negative\;control\right)}{\left(OD\;positive\;control-OD\;negative\;control\right)}\right)\times 100$$

### 2.6. Cytotoxicity in Vero Cells

Viable cells were quantified using the MTT colorimetric assay, which measures the reduction of the tetrazolium dye 3-(4,5-dimethylthiazol-2-yl)-2,5-diphenyltetrazolium bromide (MTT) by mitochondrial enzymes, indicative of metabolic activity [32]. Briefly, 100 μL of Vero cells (10^5^ cells per well) were seeded into 96-well culture plates containing serum-free Dulbecco’s Modified Eagle Medium (DMEM) (Aldrich Millipore-Sigma, St. Louis, MO, USA). The cells were then treated with different concentrations of *H. coagulans* AO1167B and a control. After a 30-min incubation at 37 °C and 5% CO_2_, 10 μL of MTT was added to each well, and the cells were incubated for an additional 3 h under the same conditions.

Following incubation, 100 μL of dimethyl sulfoxide (DMSO) was added to dissolve the formazan crystals. Absorbance was measured using an ELISA reader (MRX II, Dynex Technologies, Chantilly, VA, USA) at 550 nm with a 630 nm reference wavelength. The percentage of cellular viability was calculated using the following formula:

$$\%\;Viability=\left(\frac{At}{Ac}\right)\times 100$$

where *At* is the absorbance of *H. coagulans*-treated cells, and *Ac* is the absorbance of control cells. Control cells were treated with the following excipients from the bacterial capsule: calcium phosphate, magnesium stearate, and colloidal silica.

### 2.7. Human Safety Assessment

A comprehensive human safety assessment of *H. coagulans* AO1125 was conducted in a randomized, double-blind, placebo-controlled clinical trial to evaluate its safety in healthy adults. The study protocol was approved by the SF-36 Hermanos Ameijeiras Clinical and Surgical Hospital Ethics Committee, under approval number NA24ILH0038, dated 15 February 2023, the National Institute of Nutrition of Cuba, and the Cuban Ministry of Health, adhering to Good Clinical Practice protocols and the Declaration of Helsinki [33]. This trial was registered with the Cuban Public Registry of Clinical Trials (RPCEC) under the registration number RPCEC00000430.

#### 2.7.1. Study Design

**Figure 1:**
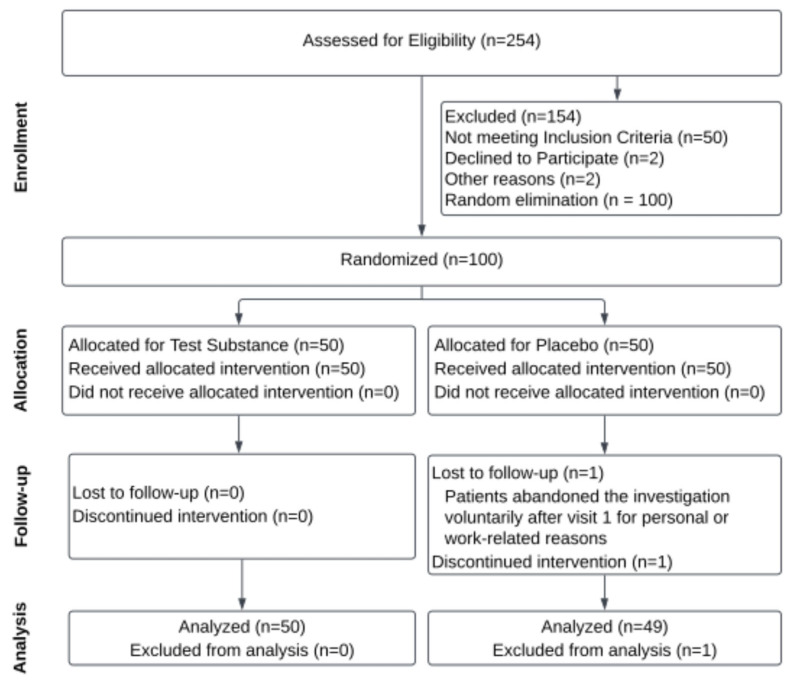
CONSORT Flow Diagram of Recruitment and Retention Throughout the Study [35].

A total of 254 patients were accepted for eligibility, 54 were excluded, and 100 healthy volunteers were randomly allocated to either the *H. coagulans* AO1167B or Placebo groups, with 50 individuals in each group. The assignment to treatment groups was randomized using the Epidemiological Analysis from Tabulated Data (EpiData 3.1) [34]. The CONSORT Flow Diagram [35] summarizing the study design and cohort participation is shown in Figure 1. Demographic and baseline characteristics are summarized in Table 2.

The required sample size was estimated with a two-sided alpha of 0.05, a 95% confidence level, a standardized mean difference of 0.75, and an 80% power.

The intervention spanned an eight-week period, during which participants in the AO1127B group received daily oral doses of the test substance, consisting of *H. coagulans* AO1167B. Each capsule contained a standardized dose of 2.5 × 10^9^ AFU (Active Fluorescent Units) of the test substance, along with excipients, including calcium phosphate, magnesium stearate, and colloidal silica, to ensure the stability and consistency of the formulation. Participants in the placebo group received capsules that contained only the excipients and were identical in appearance to the active treatment capsules to maintain blinding. All participants were instructed to take one capsule each morning, 40 min before breakfast, to standardize timing and optimize absorption in fasting conditions. Adherence was closely monitored through regular checks and participant logs, and any deviations from the dose regimen were documented to ensure compliance.

Prior to enrollment, informed consent was obtained from each participant, and strict eligibility criteria were applied to ensure that only individuals with normal hematological, clinical chemistry, and hemodynamic parameters were included. The study recruited healthy men and women aged 18 years and older, without distinction of sex or skin color, who signed the informed consent form and met the inclusion criteria. Exclusion criteria included individuals with a history of comorbidities, chronic illnesses, or any deviations from normal health parameters that could interfere with study outcomes or participant safety. Those who did not provide a signed, written informed consent were also excluded. Participants were randomly assigned to either the intervention or placebo group through simple randomization, with allocation determined using a computer-generated random number sequence to ensure unbiased group assignment.

Participants were randomized to the intervention or placebo group using simple randomization to ensure unbiased allocation. A computer-generated random number sequence was created prior to the start of the study, with odd numbers corresponding to the intervention group and even numbers to the placebo group. This randomization process ensured that each participant had an equal chance of being assigned to either group. The allocation sequence was concealed from investigators and study staff until the moment of assignment to maintain allocation blinding and minimize bias.

Following and baseline screening, the final cohort comprised 50 participants in the AO1167B group and 49 in the placebo group, reflecting 1 exclusion post-randomization. Baseline characteristics and demographic data of both groups were collected and are summarized in Table 2, demonstrating the comparability of the groups at study entry.

The participant flow, randomization process, and group allocations are summarized in the CONSORT Flow Diagram (Figure 1). This figure outlines the progression of participants through the study, including those assessed for eligibility, those excluded, those randomized into the placebo, low-dose, and high-dose groups, and those who completed or withdrew from the study. The diagram ensures transparency and provides a clear visual representation of participant handling and group assignments throughout the trial [35].

#### 2.7.2. Study Outcomes

In this safety clinical trial, the Primary Outcome was the frequency, severity, and nature of adverse events (AEs), assessed through standardized adverse event reporting forms, to evaluate the tolerability of the intervention. Secondary Outcomes included detailed assessments of clinical laboratory parameters, including biochemical markers such as creatinine, urea, alanine aminotransferase (ALAT), aspartate aminotransferase (ASAT), gamma-glutamyl transferase (GGT), total protein, albumin, glycemia, cholesterol, triglycerides, total bilirubin, and direct bilirubin. Hematological markers were also evaluated, including white blood cell count (WBC), red blood cell count (RBC), hemoglobin (HGB), hematocrit (HTC), mean corpuscular volume (MCV), mean corpuscular hemoglobin concentration (MCHC), platelet count (PLT), red cell distribution width—coefficient of variation (RDWCV), and mean platelet volume (MPV). Additional secondary outcomes included bioimpedance variables, such as weight and body mass index (BMI), and participant-reported quality of life (QoL), assessed using the SF-36 questionnaire. Together, these measures provide a comprehensive evaluation of the safety profile of *Heyndrickxia coagulans* AO1167B, while ensuring no significant adverse effects were observed across diverse physiological and quality-of-life domains.

#### 2.7.3. Safety Assessment

Safety was evaluated through monitoring of participants during the study. This included the documentation and assessment of adverse events, which were recorded in real time and categorized by severity, duration, and potential relationship to the intervention. In addition to adverse event monitoring, changes in hematological, biochemical, and hemodynamic parameters were closely tracked to identify any physiological effects of the intervention. Hematological assessments included a complete blood count (CBC) with differential to monitor for any shifts in white and red blood cell counts, hemoglobin levels, and platelet counts. Biochemical analyses covered key indicators such as liver function tests (ALT, AST), renal function markers (creatinine, BUN), and electrolyte balance, ensuring a thorough overview of metabolic stability. Hemodynamic parameters, including blood pressure and heart rate, were regularly measured to detect any cardiovascular effects.

Overall health status was evaluated using the Health Questionnaire SF-36 (version 2) [35]. This questionnaire was administered at baseline and again at the end of the study, allowing for a comparative analysis of health-related quality of life and any potential impact of *H. coagulans* AO1167B on overall wellness.

Clinical evaluations and sample collections for these parameters were conducted at two main time points: baseline (prior to the start of intervention) and at the end of the eight-week study period. These data points provided a comparative basis for assessing any significant changes in health indicators, helping to assess the safety profile of *H. coagulans* AO1167B over the course of the study.

#### 2.7.4. Sample Collection, Processing, and Data Management

Participants underwent sample collection, supplement delivery, and clinical evaluation at the beginning (week 1) and the end of the study (week 8). Samples were collected by venipuncture, then properly identified with the inclusion number, processed, and aliquoted within one hour for storage and future use. All records were maintained in a dedicated database. Access to these records was limited to study and clinical staff responsible for patient care. The Clinical Hospital Hermanos Ameijeiras (HHA) was responsible for managing the security of the information technology infrastructure.

#### 2.7.5. Clinical Determinations

Hematology parameters for obtaining standard complete blood count data were assessed through the utilization of a hematologic complex autoanalyzer XN-350 (Roche Diagnostics, Basel, Switzerland). This analysis was performed in strict adherence to the manufacturer’s guidelines, with blood samples collected in K3 EDTA tubes at the baseline (one week before the study commencement) and at the study’s conclusion (week 8).

For the evaluation of clinical chemistry parameters, a Cobas 600 modular immunochemical autoanalyzer (Roche Diagnostics) was employed. The analysis was performed on serum samples, following the manufacturer’s recommended protocols.

#### 2.7.6. Occurrence of Adverse Events Determination

The occurrence of adverse events was documented by the investigators in the case report forms for each subject adhering to the framework outlined by LeFevre et al. [31]. The relationship between each adverse event and the subject’s involvement in the study was assessed and categorized as improbable, possible, probable, or definite. Additionally, the investigators ranked the severity of each adverse event as mild (with no impediment to daily activities), moderate (resulting in partial limitations to daily activities), or severe (rendering daily activities unattainable).

#### 2.7.7. Statistical Analyses

All clinical data collected were analyzed using Statistical Package for Social Sciences (SPSS) version 23.0. Descriptive statistics were used to characterize the samples. Qualitative variables were summarized in absolute numbers and percentages, while quantitative variables were summarized as mean and standard deviation (SD) for normally distributed data. To compare differences between groups according to qualitative variables, the chi-square test (χ^2^) or Fisher’s exact test was used, while the student’s t-test was used for age and clinical, hematological, and bioimpedance quantitative variables.

The number of subjects with at least one adverse effect and the association of adverse events with study participation were compared between the H. coagulans AO1167B and placebo groups using the Fisher’s exact test. The Kappa Test was applied to evaluate the congruence in the responses to the health questionary (SF-36) in each group at both time periods (before-after). α = 0.05 was employed in all hypothesis test as significant level.

## 3. Results

### 3.1. Species and Strain Identification

On TGY agar, colonies of *H. coagulans* appeared as white to cream-colored circular colonies with smooth edges, measuring 2–3 mm in diameter after 48 h growth at 35 ± 0.5 °C. *H. coagulans* AO1167B is a Gram-positive to Gram-variable, spore-forming, motile rod measuring 3.0 to 5.5 μm in length with a mean diameter of 0.9 μm.

The MiGA analysis revealed that strain AO1167B showed as closest in homology (99.7%) to *Bacillus coagulans* CGD018 at 99% coverage. A total of 3 contigs, the longest of which was 3,317,562 bp, representing 100% of the genome of 3,320,802 bp, were analyzed, with a very low level of contamination (3.8%) and excellent quality (81%). The G + C content was determined to be 46.9779% with a G-C skew of −0.231% and an A-T skew of 0.1593%. Analysis of the assembled genome revealed a total of 3283 predicted proteins with an average length of 276.1672 amino acids. The genome has a coding density of 81.9071%.

The complete genome of *H. coagulans* strain AO1167B has been deposited with GenBank with accession number SAMN43867243 and is available for download and analysis.

**Figure 2:**
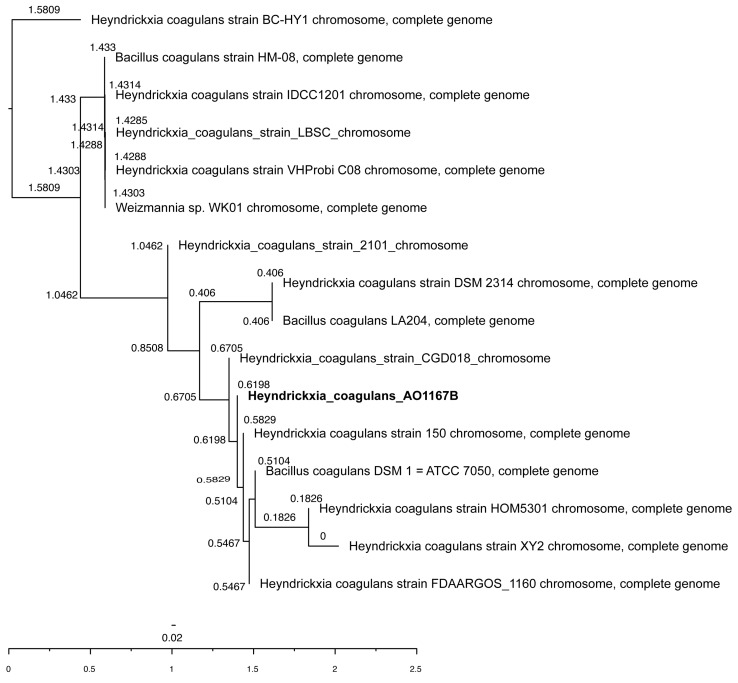
Whole genome phylogenetic analysis of *H. coagulans* AO1167B and related strains. The Neighbor-Joining tree represents the phylogenetic relationship of *H. coagulans* AO1167B with closely related strains known for probiotic properties. The tree was constructed based on whole genome sequences, with bootstrap values indicated at each node to demonstrate clustering robustness. The scale bar represents the number of substitutions per site. The tree was visualized using FigTree v1.4.4 (http://tree.bio.ed.ac.uk/software/figtree/ accessed on 4 October 2024).

Whole genome phylogenetic analysis revealed the closest strains with reported probiotic properties are CGD018 (Accession number CP051674.1), 150 (Accession number CP107276.1), DSM1 (Accession number CP008709.1), HOM5301 (Accession number CP120208.1), XY2 (Accession number CP110483.1), and FDAARGOS_1160 (Accession number CP068054.1). The result of the Neighbor Joining tree is illustrated in Figure 2.

### 3.2. Traits Associated with Probiotic Properties

#### 3.2.1. Phenotypic Traits

*H. coagulans* AO1167B demonstrated tolerance to various environmental stressors relevant to the gastrointestinal tract, underscoring its potential as a probiotic candidate. The strain exhibited resistance to lysozyme, maintaining 85.2% survival after 120 min at a concentration of 25 mg/L. When exposed to simulated gastric fluid conditions, the strain demonstrated varying survival rates across different pH levels and durations of exposure. At pH 4.1, 92.3% of cells remained viable after 20 min. As the acidity increased, survival declined to 78.7% at pH 3.0 after 40 min and to 21.7% at pH 2.1 after 60 min, indicating that the strain can persist in highly acidic stomach environments long enough to reach the intestines. Additionally, AO1167B showed high tolerance to bile salts, with 91.3% survival after 30 min of exposure to 0.5% bile salts and 82.4% survival at the higher concentration of 1.0%.

#### 3.2.2. Genotypic Properties

Genes conferring potential probiotic properties to *H. coagulans* AO1167B were systematically identified through comprehensive analysis of the annotated genome (Table 3). The genomic searches focused on identifying genes involved in mechanisms that are key to probiotic function, including stress tolerance, adhesion to intestinal epithelial cells, antimicrobial production, and modulation of the host immune response. The detailed summary of the identified genes and their associated probiotic attributes is provided in Table 3, outlining the specific contributions of these genes to the overall efficacy of *H. coagulans* AO1167B.

### 3.3. Safety Assessment of H. coagulans AO1167B

#### 3.3.1. Antimicrobial Resistance and Virulence Factors

The results in Table 4 indicate that *H. coagulans* strain AO1167B is fully susceptible to all tested antimicrobials, representing various classes: Ampicillin (beta-lactam), Chloramphenicol (phenicol), Clindamycin (lincosamide), Erythromycin (macrolide), Gentamicin and Kanamycin (aminoglycoside), Streptomycin (streptomycin), Tetracycline (tetracycline), and Vancomycin (glycopeptide).

Genomic analysis identified no “Perfect” category antimicrobial resistance (AMR) genes, indicating the absence of genes with complete sequence matches to known resistance determinants. However, seven “Strict” category AMR genes were detected, with sequence identities ranging from 31.78% to 80.41%, suggesting the presence of genes with partial homology or lower confidence matches to known AMR sequences. These genes, which are summarized in Table 5, are associated with potential resistance mechanisms but do not indicate high-risk resistance profiles. Importantly, no mobile genetic elements were found in proximity to these resistance genes.

The Virulence Factor Database analysis revealed four significant sequences in *H. coagulans* AO1167B: *narH* (nitrate reductase subunit beta), *capB* (poly-gamma-glutamate synthesis), *gndA* (NADP-dependent phosphogluconate dehydrogenase), and *wbtL* (glucose-1-phosphate thymidylyl-transferase), all located on the chromosome.

Genome sequence analysis of *H. coagulans* AO1167B identified two genes encoding enzymes involved in biogenic amine production: arginine decarboxylase (EC 4.1.1.19) and lysine decarboxylase (EC 4.1.1.18). The first arginine decarboxylase gene was located on contig 1 (positions 883,091 to 881,622, negative strand) with a length of 1470 bp. The second gene, encoding both arginine decarboxylase (EC 4.1.1.19) and lysine decarboxylase (EC 4.1.1.18), was identified also on contig 1 (positions 3,075,431 to 3,076,906, positive strand) with a length of 1476 bp. Notably, no evidence of mobile elements was found in the flanking regions of these antimicrobial resistance genes.

#### 3.3.2. Hemolytic Activity and Cytotoxicity

**Figure 3:**
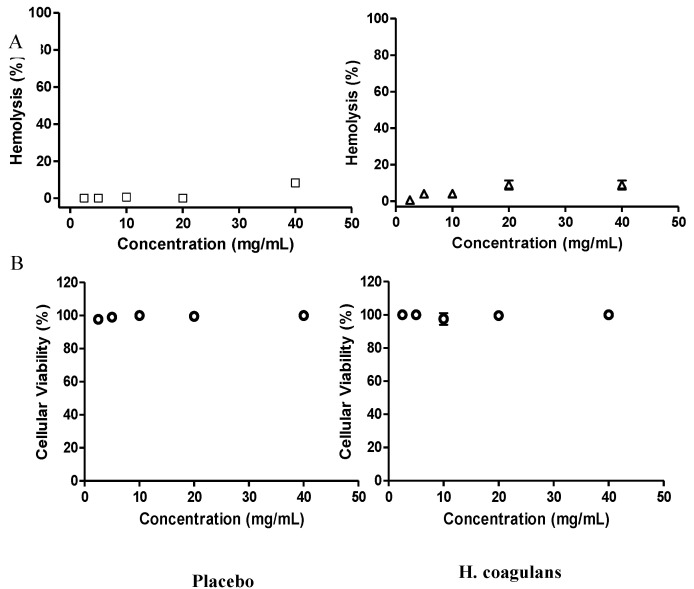
Cytotoxicity of H. coagulans AO1167B. (**A**) Hemolysis induced by H. coagulans AO1167B on 1% of human erythrocytes solution for 1 h. The hemolytic activity was expressed as % of hemoglobin (hemolysis %). (**B**) Percent of viability induced by H. coagulans AO1167B in Vero cells. The cytotoxic activity was expressed as % of viability. Error bars represent the standard error (SE) of the mean, calculated from three independent replicates.

The hemolytic potential of *H. coagulans* AO1167B was evaluated using a standardized hemolysis assay [31]. Hemoglobin release was quantified at 490 nm, with PBS serving as the negative control and distilled water as the positive control, inducing complete hemolysis. A two-tailed *t*-test [36] was conducted to compare the hemolysis levels between the *H. coagulans* AO1137B-treated group and the negative control (untreated cells). The results showed no significant difference in hemolysis levels (*p* > 0.05), supporting that *H. coagulans* AO1137B does not cause red blood cell lysis (Figure 3A).

The cytotoxicity of *H. coagulans* AO1167B was assessed using the MTT assay, which measures metabolic activity through the reduction of tetrazolium dye to formazan [32].

Similarly, a one-way ANOVA [37] was performed to assess cell Vero cell viability among the *H. coagulans* AO1137B-treated group, untreated control, and positive control. There was no statistically significant reduction in cell viability in the *H. coagulans* AO1137B group compared to the untreated control (*p* > 0.05), indicating that *H. coagulans* AO1137B is non-cytotoxic (Figure 3B)

#### 3.3.3. Clinical and Hematological Determinations

Clinical and hematological assessments conducted throughout the study revealed that all participants remained within normal ranges for the parameters evaluated (Table 6), indicating no clinically relevant abnormalities. However, significant differences were observed in some specific clinical and hematological parameters across the study groups. These differences, while statistically significant, did not exceed clinically acceptable thresholds and were consistent with expected physiological variations under the study conditions.

The analysis of independent samples showed, at baseline, that only cholesterol levels were statistically significant (*p* = 0.020). By day 60, significant changes were observed in albumin (*p* < 0.01), total triglycerides (*p* = 0.031), and total bilirubin (*p* = 0.028) in both study groups. Significant differences were found at both baseline (ALAT *p* = 0.008, ASAT *p* < 0.01) and day 60 (ALAT *p* = 0.047, ASAT *p* < 0.01).

In the *H. coagulans* group, a student’s *t*-test for related samples showed significant decreases in creatinine (*p* = 0.032), albumin (*p* < 0.01), and MVC (*p* = 0.003). Significant increases were found in ASAT (*p* < 0.01), triglycerides (*p* = 0.001), total bilirubin (*p* = 0.010), MCHC (*p* < 0.01), and MPV (*p* = 0.040).

In the placebo group, significant increases were detected in urea (*p* = 0.012), GGT (*p* = 0.026), HTC (*p* = 0.001), and MVC (*p* < 0.01). Significant reductions were found in ALAT (*p* = 0.002), ASAT (*p* < 0.01), cholesterol (*p* < 0.01), total bilirubin (*p* = 0.002), and direct bilirubin (*p* = 0.001).

Bioimpedance measurements of weight and BMI showed no significant differences between study groups when analyzed using the *t*-test for independent and related samples.

#### 3.3.4. Adverse Events

The study included 99 individuals, with 49 in the placebo group and 50 in the *H. coagulans* group. Adverse events (AEs) evaluated were abdominal/gastrointestinal discomfort, acne rosacea, anxiety, depression, joint pain, bronchitis, excision of birthmarks, bruises, carotid stenosis, cataract surgery, chondrocalcinosis, cystitis, dental pain, diarrhea, dizziness, nausea, oedema, gas, general aches, genital herpes, headache, hemorrhoids, infection, inflamed prostate, migraine, mouth ulcer, muscle discomfort, nasal obstruction, orthopedic pain, pain after capsule consumption, palpitations, radio-infiltration (shoulder), rhinitis, sore throat, tracheitis, insomnia, vaginal dryness, vagal seizures during or after blood sampling, vitamin D deficiency, and constipation.

Most participants (86.9%, n = 86) did not experience any AEs (Table 7). Among them, 80% were in the group receiving the test substance (n = 40) and 93.9% were in the placebo group (n = 46). There was a significant difference in the incidence of AEs between the treated and placebo groups (*p* = 0.04) (Table 7).

Three AE cases occurred in the Placebo group: two reported looser stools, and the third reported oedema, which coincided with a new dengue infection, likely linked to the virus. Among those taking the test substance capsules, 10 individuals (20%) experienced mild AE. These included gastrointestinal pain or discomfort (n = 2), gas (n = 5), headache (n = 1), and constipation (n = 2).

The study comprised 62 females and 37 males. Most AE cases were reported by females (10/62) compared to males (3/37). One female (7%) from the Placebo group experienced moderate intensity AE coinciding with a dengue infection debut; others reported mild effects that lessened over treatment days without hindering daily activities.

#### 3.3.5. Health Questionnaire Analysis

The SF-36 questionnaire, specifically questions 1, 6, 7, and 11, focusing on physical and emotional health status, was analyzed to evaluate changes over time in both study groups. Responses to these questions demonstrated highly similar patterns between the baseline and follow-up assessments, with nearly perfect agreement reflected by a Kappa coefficient approaching 1 (Kappa~1, *p* = 0.000). This strong concordance indicates remarkable consistency in participants’ perceptions of their physical and emotional health over the course of the study (Table 8).

## 4. Discussion

### 4.1. Species and Strain Identification

The phenotypic and genomic characterization of *H. coagulans* AO1167B highlights its close relationship to probiotic strains of *H. coagulans*. Its colony morphology, spore-forming ability, and motility are consistent with traits reported for strains such as CGD018 and DSM1, supporting its classification within the species. Genomic analysis revealed a 99.7% homology to *B. coagulans* CGD018 and high-quality assembly with 100% genome coverage and minimal contamination (3.8%). AO1167B’s coding density (81.91%) and predicted 3283 proteins indicate functional richness comparable to other strains, and its stable genomic architecture (46.98% G + C content) further underscores its potential probiotic efficacy.

The phylogenetic analysis (Figure 2) situates AO1167B within a clade of well-characterized strains, including CGD018, DSM1, HOM5301, and FDAARGOS_1160, which have demonstrated probiotic properties. AO1167B’s clustering with these strains underscores its evolutionary relatedness and functional potential. The tree also reveals subtle evolutionary divergence, suggesting unique strain-specific traits that may enhance its use for probiotic applications. For example, its close genomic and phylogenetic relationship to CGD018 and DSM1, both of which exhibit gastrointestinal resilience and health benefits, highlights AO1167B’s suitability for targeted formulations.

Together, these findings confirm AO1167B as a robust candidate for probiotic development. Its genetic proximity to other well-studied *H. coagulans* strains, combined with its distinct genomic features, positions it as an ideal strain for further exploration in clinical and functional studies.

### 4.2. Traits of H. coagulans AO1167B Associated with Probiotic Properties

#### 4.2.1. Phenotypic Traits

*H. coagulans* AO1167B exhibited characteristics under experimental conditions that are consistent with the properties of a candidate for probiotic development [38]. The strain demonstrated significant tolerance to bile salts and gastric acidity, key factors for survival and persistence within the gastrointestinal environment. Additionally, AO1167B displayed resistance to lysozyme, an antibacterial enzyme present in human secretions, further supporting its potential for colonization and stability within the host system. Genomic analysis revealed the absence of transmissible plasmids, minimizing concerns regarding horizontal gene transfer [39]. Moreover, in vitro assays confirmed a lack of antimicrobial resistance, reinforcing the strain’s safety profile for probiotic use without contributing to antimicrobial resistance development.

Our findings regarding *H. coagulans* AO1167B align with and extend the current literature on the probiotic potential of this species. Recent studies have emphasized the diverse roles of *H. coagulans* strains in enhancing immunity and addressing metabolic disorders through microbiota modulation [40,41]. The introduction of any new strain, however, necessitates robust safety and efficacy evaluations. In this study, the strain AO1167B underwent comprehensive in vitro and in vivo characterizations to establish its suitability for use as a food or nutritional supplement.

Comparing our results to previously published data, the morphological, cultural, and genotypic analyses confirmed that AO1167B is a pure culture and closely related to *H. coagulans* strains [42,43]. These findings are consistent with prior reports highlighting the genetic stability and taxonomic alignment of probiotic *H. coagulans* strains.

One of the critical determinants of probiotic functionality is the ability to withstand gastrointestinal stress. In this regard, AO1167B exhibited remarkable resilience to environmental stressors. Specifically, its tolerance to lysozyme (85.2% survival at 25 mg/L after 120 min) compares favorably with other strains of *H. coagulans* previously reported in the literature [44]. Similarly, *H. coagulans* AO1167B demonstrated robust gastric fluid tolerance, maintaining high viability at pH levels ranging from 4.1 (92.3% survival after 20 min) to 3.0 (78.7% survival after 40 min). The strain’s decline in survival at pH 2.1 (21.7% survival after 60 min) reflects the extreme stress of such acidic conditions, which nonetheless underscores the strain’s potential to reach the small intestine in a viable state, as reported for other well-characterized probiotics [45].

In addition to acid tolerance, AO1167B demonstrated significant bile salt resistance, with survival rates of 91.3% and 82.4% after 30 min in 0.5% and 1.0% bile salts, respectively. These results align with the bile tolerance profiles of probiotic strains in the genus, which are essential for colonization and activity in the small intestine [46].

Safety assessments are pivotal for evaluating new probiotics, and *H. coagulans* AO1167B exhibited no cytotoxic effects on Vero cells and no hemolytic activity in vitro. These findings reinforce its safety profile and are consistent with other studies where *H. coagulans* strains have been deemed safe for human consumption [17,47].

Based on these findings, *H. coagulans* AO1167B exhibits properties comparable to or exceeding those reported for other strains in the literature. Its tolerance to gastrointestinal conditions and lack of cytotoxicity highlights its potential as a safe and effective probiotic candidate. Further studies, including clinical evaluations, are warranted to validate these findings and assess the strain’s safety and efficacy in vivo.

#### 4.2.2. Genotypic Traits

Previous studies have underscored the importance of specific genes in conferring probiotic properties to bacterial strains, particularly within the *Heyndrickxia coagulans* species and related genera [38,48,49]. Genomic analyses have revealed that traits such as acid and bile tolerance, adhesion, stress resistance, and immunomodulation are governed by distinct genetic components that enable survival and functionality in the gastrointestinal tract. For example, bile salt hydrolases and ATP synthase subunits are critical for stress adaptation [50], while surface-associated proteins like pilins and enolases enhance adhesion to host epithelial cells [51]. Additionally, genes linked to antioxidant defense [52], detoxification [53], and short-chain fatty acid (SCFA) production further augment probiotic efficacy [54]. Against this backdrop, the genome of *H. coagulans* AO1167B offers a robust repertoire of genes that collectively underpin its potential as a probiotic strain.

The genomic analysis of *H. coagulans* AO1167B possesses a panel of genes that support acid and bile tolerance, key traits for probiotic survival in the harsh gastrointestinal environment. Genes such as *atpA* and *atpB* (ATP synthase subunits) and arcD1 (arginine/ornithine antiporter) enable the strain to maintain pH homeostasis and cellular energy balance under stress conditions [55,56]. Similarly, bsh and bshB (bile salt hydrolases) contribute to bile deconjugation, enhancing survival and interaction with the host’s bile metabolism [57].

Adhesion and aggregation capabilities, which are critical for effective gut colonization, are supported by genes like *spaCBA* (pilin), *eno* (enolase), and *ugtP* (glucosyltransferase) [58]. These genes facilitate attachment to intestinal epithelial cells and biofilm formation, improving persistence and probiotic efficacy [59]. Additionally, *mntH* (divalent metal cation transporter) and *tuf* (elongation factor Tu) enhance cellular aggregation and stability, further bolstering the strain’s resilience in the gut [60].

Antioxidant defense mechanisms encoded by genes such as *trxA* (thioredoxin) and *katE* (catalase) enable AO1167B to neutralize reactive oxygen species, protecting itself and contributing to host gut health [61]. Stress tolerance and detoxification are further reinforced by genes like *phoPR* (two-component response regulator) and *sigB* (sigma factor B), which aid in adaptation to environmental stresses, including storage conditions and gastrointestinal transit [62]. Additionally, detoxification genes such as *arsC* (arsenate reductase) and *cadA* (cadmium-transporting ATPase) suggest the strain’s ability to mitigate harmful substances, enhancing its robustness and safety [63].

Carbohydrate metabolism genes such as *xylA* (xylose isomerase) and *galT* (UDP-glucose--hexose-1-phosphate uridylyl transferase) equip AO1167B to utilize diverse sugars, supporting growth and energy production in the gut environment [64]. The presence of *bgaB* (beta-galactosidase) highlights its ability to ferment lactose and other prebiotic sugars, potentially benefiting the host microbiota. Additionally, *ackA* (acetate kinase) and *pta* (phosphotransacetylase) suggest a capacity for SCFA production, particularly acetate, which supports gut barrier integrity and systemic metabolic regulation [65].

The strain also exhibits immune-modulatory potential, as evidenced by genes such as *groEL* (chaperonin GroEL) and *ctkA* (serine/threonine protein kinase), which have been linked to interactions with the host immune system [66]. Moreover, genes like hag (flagellin) and *cheA* (chemotaxis protein) enable motility and biofilm formation, enhancing its ability to colonize and interact dynamically with the host microbiota [67].

Finally, the strain’s ability to synthesize essential vitamins, including biotin, cobalamin (B12), and thiamine, is supported by genes such as *bioB* (biotin synthase), *cobA* (adenosylcobalamin synthase), and *thieE* (thiamine phosphate synthase). These biosynthetic capabilities not only add nutritional benefits for the host but also distinguish *H. coagulans* AO1167B as a multifunctional probiotic candidate.

In summary, the genome of *H. coagulans* AO1167B encodes a comprehensive suite of genes that support its probiotic potential. These genes collectively enable acid and bile tolerance, adhesion, SCFA production, stress resistance, and immune modulation, while also contributing to nutritional benefits. The genetic insights into AO1167B underscore its promise as a robust and versatile probiotic strain, suitable for clinical and functional applications aimed at improving gut health and overall well-being.

### 4.3. Antimicrobial Resistance

The presence of resistance-associated genes, including *vanH*, *vanY*, *vanT*, *vanW*, *qacG*, *qacJ*, and *acrB*, in *H. coagulans* AO1167B requires careful evaluation to ensure the strain’s safety for probiotic use (Table 5). This assessment is supported by genomic, phenotypic, and regulatory considerations.

Genomic analysis reveals that the putative glycopeptide resistance genes (*vanH*, *vanY*, *vanT*, and *vanW*) in *H. coagulans* AO1167B are chromosomally encoded and not associated with mobile genetic elements such as plasmids or transposons. Furthermore, no plasmids were detected in the genome, strongly suggesting that these genes are intrinsic and unlikely to be transferred horizontally. Sequence analysis against the CARD database indicates low homology (<36%) to canonical glycopeptide resistance clusters, supporting the interpretation that these genes are analogs or non-functional variants. Phenotypic testing through Kirby–Bauer disk diffusion confirms that *H. coagulans* AO1167B is sensitive to vancomycin, indicating that these genes are not expressed under normal conditions (Table 4). These findings align with regulatory precedents, as intrinsic glycopeptide resistance has been deemed acceptable in probiotic strains, including *Lactobacillus* spp., provided there is no risk of resistance transfer [68,69].

The efflux-related genes *qacG* and *qacJ* encode small multidrug resistance (SMR) proteins that facilitate the efflux of quaternary ammonium compounds (QACs), commonly found in disinfectants. These genes are intrinsic and contribute to environmental adaptation rather than resistance to clinically significant antibiotics. Similar efflux systems are present in widely used probiotics and have not been associated with safety concerns [70]. The *acrB* gene encodes a Resistance-Nodulation-Division (RND) efflux pump that likely aids *H. coagulans* in surviving stressful environments, such as bile salt exposure in the gastrointestinal tract. Efflux systems like *acrB* are widespread in bacteria, including probiotics, and are considered part of their natural physiology [71]. Sequence analysis against CARD revealed moderate homology (<50%) to known multidrug resistance-associated efflux pumps, further suggesting that these genes are intrinsic and unrelated to clinical resistance mechanisms. The absence of plasmids in the genome further supports the chromosomal nature of these efflux genes, minimizing the likelihood of horizontal gene transfer. Phenotypic testing confirms that *H. coagulans* AO1167B is sensitive to antibiotics commonly affected by RND efflux pumps, further supporting its safety profile.

According to regulatory guidelines from EFSA and FDA, intrinsic resistance genes that lack horizontal transfer potential and clinical relevance are acceptable in probiotics (EFSA, 2018). The intrinsic nature of the resistance-associated genes in *H. coagulans* AO1167B, combined with phenotypic evidence of antibiotic susceptibility, low sequence homology to clinically relevant resistance genes, and the absence of plasmids, demonstrates that these genes do not compromise the strain’s safety or efficacy. Similar precedents exist for other probiotics, such as *Lactobacillus* and *Bifidobacterium* species, where intrinsic resistance genes have been deemed safe due to their lack of horizontal transfer potential [72].

The intrinsic resistance-associated genes identified in *H. coagulans* AO1167B reflect natural bacterial adaptations to environmental pressures rather than clinically significant resistance mechanisms [73]. Genomic and phenotypic evidence, coupled with regulatory precedents, supports the safety of this strain. Therefore, the presence of these genes does not pose a safety risk, and *H. coagulans* AO1167B can be confidently considered safe for human consumption.

### 4.4. Virulence Genes

Using the Virulence Factor Database (VFDB), a comprehensive analysis of the genome of *H. coagulans* AO1167B identified four sequences homologous to known virulence-associated genes: *narH*, *capB*, *gndA*, and *wbtL*. These genes are widely distributed among various bacterial species and are typically associated with fundamental metabolic or structural functions rather than pathogenicity in non-pathogenic or environmental strains like *H. coagulans*.

The *narH* gene plays a central role in bacterial metabolism by facilitating the reduction of nitrate to nitrite, thereby enabling bacteria to thrive under anaerobic conditions and utilize nitrate as an alternative electron acceptor [74], maintaining redox balance, and enhancing metabolic flexibility in anaerobic conditions [75].

The capB gene synthesizes capsular polysaccharides in bacteria [76]. These polysaccharides enhance bacterial survival and pathogenesis by shielding the bacterium from host defenses, promoting adherence to host tissues, and facilitating colonization and infection. They also aid in bacterial survival during gastrointestinal transit and promote colonization of the intestinal mucosa. Additionally, capsular polysaccharides promote adherence to gut epithelial cells, crucial for probiotic bacteria to establish and maintain beneficial interactions with the host [77].

The *gndA* gene encodes a protein that converts 6-phosphogluconate to gluconate-6-phosphate, a critical step in the pentose phosphate pathway [78]. This pathway supports metabolic flexibility in probiotic bacteria, potentially enhancing gut health benefits such as improved colonization and energy production [79].

The wbtL gene encodes a glycosyltransferase that synthesizes bacterial cell surface polysaccharides, particularly the O antigen of lipopolysaccharides (LPS) in gram-negative bacteria [80]. In pathogens, wbtL contributes to assembling and modifying the LPS O antigen, which masks immunogenic components and aids in evading host immune responses [81]. Although less studied in probiotic bacteria, similar glycosyltransferase enzymes could potentially modify surface polysaccharides, influencing interactions with the host immune system or gut microbiota and contributing to probiotic functionality [82,83].

*H. coagulans* AO1167B showed no cytotoxicity to Vero cells and no hemolytic activity against erythrocytes in vitro, consistent with previous findings on *B. coagulans* CGI314 under similar conditions [84]. These results affirm the strain’s favorable safety profile for potential probiotic applications.

### 4.5. Bioactive Amines

Microorganisms synthesize a wide range of metabolites that can have both beneficial and harmful effects on human health. Among these are amino acid derivatives produced during bacterial growth and fermentation, which can interact with human physiology in diverse ways, exhibiting health-modulating potential [85]. This category includes bioactive compounds such as biogenic amines (BAs), which are associated with adverse health effects and implicated in several pathogenic conditions [85]. Consuming foods with high levels of BAs poses a health risk, as these compounds can trigger symptoms such as headaches, heart palpitations, vomiting, diarrhea, and hypertensive crises [86]. The toxic effects of BAs, however, depend on factors such as the specific type of BA, individual sensitivity or allergies, and the concurrent use of monoamine oxidase inhibitors or alcohol, which interfere with the enzymatic detoxification of exogenous BAs by amine oxidases [87].

The identified bioactive amine genes coding form arginine decarboxylase (EC 4.1.1.19) and lysine decarboxylase (EC 4.1.1.18) are important for the metabolism of *H. coagulans* AO1167B. The presence of two genes suggests that this strain can produce biogenic amines, which are critical for several metabolic processes. Arginine decarboxylase converts arginine to agmatine, a precursor in polyamine biosynthesis, which is essential for cell growth, gene regulation, and stress response mechanisms [88]. Similarly, lysine decarboxylase catalyzes the production of cadaverine from lysine, a biogenic amine that plays a role in maintaining cellular homeostasis, particularly in acid stress resistance [88,89]. These metabolic activities are likely crucial for the strain’s survival and adaptation, particularly in the harsh conditions of the gastrointestinal tract, enhancing its probiotic potential.

### 4.6. Clinical Study

Safety assessments are critical when introducing any new strain of *H. coagulans* human consumption, particularly given its potential for widespread use in food and nutritional supplements [90,91,92]. While *H. coagulans* strains have been increasingly recognized for their health-promoting properties, including immunomodulation and metabolic benefits [49,93], rigorous safety studies are necessary to ensure their suitability for human consumption. These evaluations typically encompass in vitro and in vivo analyses to assess cytotoxicity, hemolytic activity, and resistance to gastrointestinal conditions, as well as the absence of transferable antibiotic resistance genes [3,4]. Such studies not only confirm the strain’s non-pathogenicity but also provide essential data for regulatory compliance and consumer safety. Given the variability among strains, including differences in metabolic activities and the potential for producing harmful byproducts such as biogenic amines, strain-specific investigations are indispensable [11,94]. These assessments provide a foundation for confidently integrating *H. coagulans* into probiotic formulations targeted at human health.

#### 4.6.1. Demographic and Safety Overview

Demographic characteristics, study duration, and product compliance were comparable between the *H. coagulans* AO1167B and placebo groups. Clinical and hematological assessments consistently remained within normal parameters throughout the study, affirming participant health and confirming the safety of *H. coagulans* AO1167B when administered at the recommended doses for healthy adults. These findings support its potential as a candidate for probiotic development (see Table 6).

The minor differences observed in clinical parameters between the treatment and placebo groups are consistent with the normal variations typically seen among healthy individuals. These differences are likely influenced by standard metabolic processes and the demographic composition of the study population [95]. The study cohort included 62 female participants, evenly distributed between the *H. coagulans* and placebo groups, with 32 females in each group. The notable increase in hepatic enzymes observed may be linked to the predominance of females in both cohorts, with a mean age of 43 years. Fluctuations in liver enzyme levels throughout the menstrual cycle, potentially affected by progesterone, can also vary depending on age and BMI [96].

Serious adverse events (SAEs) linked to probiotics have been reported in rare cases and include systemic infections, gastrointestinal complications, skin reactions, endocarditis, gene transfer to normal microbiota, metabolic disturbances, and immune stimulation [97]. The potential for such adverse outcomes underscores the importance of rigorous safety evaluation in early-phase clinical trials. In this Phase 1 trial, safety was the primary outcome, assessed primarily through the monitoring and reporting of adverse events. Importantly, no serious adverse events were observed in our study population during the intervention period, supporting the safety and tolerability of *H. coagulans* AO1167B under the conditions tested.

Most adverse events reported were mild, transient, and self-limiting, affecting only a small percentage of participants in the treated group. These findings align with those of Majeed et al., who evaluated the safety of *H. coagulans* MTCC 5856 at a similar dose (2 × 10^9^ colony-forming units/day) over a 30-day period in healthy individuals and reported no serious safety concerns [98]. The absence of serious adverse events in both our study and comparable research strengthens the evidence that *H. coagulans* strains, including AO1167B, demonstrate a favorable safety profile.

The lack of serious adverse events observed in our study is particularly noteworthy given the unique considerations associated with probiotics as live microorganisms. Unlike inert supplements or medications, probiotics possess the potential for infectivity, in situ toxin production, or unintended metabolic interactions. *H. coagulans* AO1167B demonstrated no such adverse effects, supporting its safety as a potential candidate for probiotic development.

Moreover, the mild adverse events observed in our study were comparable in frequency and severity between the treated and placebo groups, further emphasizing the safety of AO1167B. The robust safety profile observed in this trial, combined with the strain’s demonstrated ability to survive under gastric and bile conditions and its lack of transferable antibiotic resistance genes, positions *H. coagulans* AO1167B as a promising candidate for future probiotic development. These findings provide a strong foundation for advancing AO1167B to larger and more diverse clinical studies, where its efficacy and broader safety profile can be more fully evaluated.

#### 4.6.2. Limitations of the Study

This study has several limitations that warrant discussion. One key limitation is the absence of a positive control group, which could have served as a benchmark to better evaluate the effects of the probiotic candidate. As a Phase I clinical trial, the primary focus was on assessing the safety profile of the probiotic candidate rather than its efficacy. Consequently, design elements such as the inclusion of a positive control group and a detailed investigation into the probiotic’s impact on the participants’ gut microbiome were not part of this preliminary study. These aspects will be more appropriately addressed in future efficacy-oriented trials, which could expand on these findings to provide a more thorough understanding of the probiotic’s potential benefits and mechanisms.

The findings of this Phase 1 safety trial are primarily applicable to individuals with demographic and clinical characteristics similar to the study population. The trial was specifically designed with sufficient statistical power to assess the safety and tolerability of *H. coagulans* AO1167B, and the sample size was adequate to detect adverse events with a moderate incidence rate. These results provide robust evidence of safety under controlled clinical conditions. However, generalization to broader populations is limited by the study’s early-phase nature and specific inclusion and exclusion criteria, which may not represent the full diversity of potential users. Additionally, the trial was conducted in a controlled clinical environment, and the observed safety outcomes may differ when the intervention is used in real-world settings where factors such as age, health status, or dietary habits may vary.

While this trial establishes a strong safety profile for *H. coagulans* AO1167B, further studies with larger and more diverse populations across different healthcare settings are needed to confirm the generalizability of these findings. These future studies will help determine how the strain performs in real-world conditions and across various demographic groups.

Another limitation is that the intestinal viability of *H. coagulans* AO1167B was not directly assessed at the end of the study. However, the safety profile observed is supported by multiple factors: (1) in vitro assays demonstrated the strain’s resilience to bile and gastric acid, indicating its ability to survive gastrointestinal conditions; (2) previous studies with similar strains have shown high viability throughout the digestive tract, further supporting its in vivo resilience [6,99]; and (3) the absence of adverse effects across participants suggests that any potential reduction in viability, if present, did not compromise safety. Together, these findings strongly support that the observed safety profile is attributable to the strain’s inherent safety rather than diminished viability.

Additionally, the epidemiological context in Cuba during the study, including outbreaks of dengue fever and respiratory infections, may have influenced the results. Another limitation was the reliance on participants’ self-reported benefits using the test-substance capsule. All participants reported overall improvement following treatment. These subjective outcomes will be addressed in future studies using structured questionnaires to provide more standardized and quantifiable data.

## 5. Conclusions

The rigorous evaluation of microorganisms intended for nutritional supplementation, as demonstrated in this study, remains a cornerstone of scientific inquiry into probiotic safety and efficacy. Over a 60-day period, participants consumed daily doses of *H. coagulans* AO1167B (2.5 × 10^9^ CFU per capsule) with excellent tolerance and no serious adverse events reported. These results underscore the strain’s strong safety profile and its potential as a probiotic candidate for incorporation into food products and dietary supplements.

The findings of this study suggest that *H. coagulans* AO1167B holds promise for improving gut health and overall wellness, making it a viable option for promoting microbial balance and digestive health. Its ability to withstand gastrointestinal conditions, including exposure to gastric acid, bile salts, and lysozyme, further supports its functional resilience. Genomic analyses confirmed the absence of plasmids, virulence factors, hemolytic activity, and harmful secondary metabolite production, ensuring compliance with regulatory safety standards. These characteristics, combined with its demonstrated tolerability and safety, make AO1167B a compelling candidate for probiotic applications.

To enhance its application as a reliable probiotic, ongoing genomic screening should be conducted to ensure the absence of virulence and antibiotic resistance genes. Incorporating good manufacturing practices (GMP) can further mitigate contamination risks during production. Preclinical and clinical studies should focus on optimizing dosing to balance safety and efficacy, while long-term clinical trials in diverse populations can provide deeper insights into host-microbiome interactions and confirm its health benefits. Additionally, advances in genome editing and strain optimization could further refine AO1167B’s safety and functionality, solidifying its role as a dependable probiotic supplement for human health.

## Figures and Tables

**Table 1:** Biogenic and nitric oxide coding Genes in the genome of *H. coagulans* AO1167B.

Gene	Enzyme	Product
*hdcA*	Histidine decarboxylase	Histamine
*tdcA*	Tyrosine decarboxylase	Tyramine
*speC*	Ornithine decarboxylase	Putrescine
*ldc*	Lysine decarboxylase	Cadaverine
*aguA*	Agmatine ureohydrolase	Agmatine
*aguD*	Agmatine deiminase antiporter	Agmatine
*speE*	Spermidine synthetase	Spermidine
*speF*	Spermine synthetase	Spermine
*speG*	Spermidine synthetase	Spermidine
*tyrDC*	Tyrosine decarboxylase	Phenylethylamine
*tph*	Tryptophan hydroxylase	Serotonin
*aadc*	Aromatic amino acid decarboxylase	Serotonin
*tbh*	Tyramine β-hydroxylase	Octopamine
*tam*	Tryptamine synthase	Tryptamine
*sufI*	Copper-containing nitrite reductase	Nitric oxide
*nasD*	Nitrite reductase	Nitric oxide

**Table 2:** Distribution of cases according to demographics parameters.

Demographic Variables		*H. coagulans* AO1167B Cohort (*n* = 50)		Placebo Cohort (*n* = 49)		*p* Value
		Number	%	Number	%	
Sex	Female	31	62	31	63.3	0.896 ^a^
	Male	19	38	18	36.7	
Age (years) Median ± SD		42.82 *±* 16.3		44.43 *±* 14.74		1.0 ^b^
BMI Median ± SD		23.20 *±* 3.93		23.63 *±* 4.17		0.764 ^a^

^a^ Fisher test; ^b^ *t*-test.

**Table 3:** Genomic Overview of Probiotic-Associated Genes in *H. coagulans* AO1167B.

Gene	Encoded Protein
Acid tolerance	
*atpA*	ATP synthase subunit alpha
*atpB*	ATP synthase subunit beta
*ldh1*	L-lactate dehydrogenase 1
*ldhD*	D-lactate dehydrogenase
*pgi*	Glucose-6-phosphate isomerase
*groL*	60 kDa chaperonin
*cspB*	Cold shock protein
*teaD*	TRAP-T-associated universal stress protein
*ald*	Alanine dehydrogenase
*gabD*	Succinate-semialdehyde dehydrogenase
*fdhD*	formate dehydrogenase
*pgi*	Glucose-6-phosphate isomerase
*atpB*	ATP synthase subunit B
Acid/bile tolerance	
*arcD1*	Arginine/ornithine antiporter ArcD1
*iPGM*	bisphosphoglycerate-independent phosphoglycerate mutase
*argF*	Ornithine carbamoyltransferase
*gpmI*	2,3-bisphosphoglycerate-independent phosphoglycerate mutase
*argR*	Arginine repressor
Adhesion and aggregation	
*eno*	Enolase
*mntH*	Manganese transferase/Divalent metal cation transporter
*ywgD*	Tyrosine-protein kinase
*tpiA*	Triosephosphate isomerase
*ugtP*	Glucosyltransferase
*tuf*	Elongation factor Tu
*spaCBA*	Pilin
Antioxidant defense	
*trxA*	Thioredoxin
*katE*	Catalase
Detoxification	
*arsC*	Arsenate reductase
*bsh*	Bile salt hydrolase
*cadA*	Cadmium-transporting ATPase
*hsp18*	18 kDa heat shock protein
Bile tolerance	
nagB	Glucosamine-6-phosphate deaminase
pyrG	CTP (cytidine triphosphate synthetase) synthase
nagB	Glucosamine-6-phosphate deaminase
bshB	Bile salt hydrolase
Biofilm formation/Adhesion/Chemotaxis	
*luxS*	Lyase
*cheA*	Chemotaxis protein CheA
*srfA*	Surfactin synthetase
*hag*	Flagellin
*motB*	Motility protein
*manX*	mannose-specific EIIAB component
Carbohydrate metabolism	
*galT*	UDP-glucose--hexose-1-phosphate uridylyl transferase
*nagA*	Glucosamine-6-phosphate deaminase
*bgaB*	Beta-galactosidase
*agaA*	alpha galactosidase
*bbmA*	intracellular maltogenic amylase
*gntR*	Xylose utilization operon
*xylA*	Xylose isomerase
*galE*	UDP-glucose 4-epimerase
Cell wall remodeling	
*pbp*	Penicillin-binding protein
*murA*	UDP-N-acetylglucosamine enol pyruvyl transferase
Immune modulation	
*hemA*	Glutamyl-tRNA reductase
*groEL*	Chaperonin GroEL
*magl*	Monoacylglycerol lipase
*ctkA*	Serine/threonine protein kinase
Metabolism	
*feoB*	Ferrous ion transporter
*ldh*	L-lactate dehydrogenase
*lacA*	Beta-galactosidase
*glnA*	Glutamine synthetase
*UreA*	Urease
*methH*	Methionine synthase
*phoB*	Phosphate regulon protein
*pstS*	Phosphate-binding protein
*clpP*	Protease ClpP
*purA*	Adenylosuccinate synthetase
SCFA (acetate) production	
*ackA*	Acetate kinase
*pta*	Phosphotransacetylase
Stress tolerance/Response	
*phoPR*	Two-component response regulator
*sigH*	Sigma factor H
*sigB*	Sigma factor B
*clpL*	Stress response protein
*msrA*	Methionine sulfoxide reductase
*acoA*	Oxidoreductase
*yvbW*	Amino acid permease
*emrY*	Multidrug resistance protein
*TeaD*	Universal stress protein
Vitamin biosynthesis	
*cobA*	Adenosylcobalamin synthase
*fadA*	3-ketoacyl-CoA thiolase @ Acetyl-CoA acetyltransferase
*fadD_1*	Long-chain-fatty-acid-CoA ligase
*bioB*	Biotin synthase
*hpt*	Hypoxanthine-guanine phosphoribosyltransferase
*dfrA*	Dihydrofolate reductase
*thyA2*	Thymidylate synthase
*serA*	D-3-phosphoglycerate dehydrogenase
*dagK*	Diacylglycerol kinase
*thieE*	Thiamine phosphate synthase

**Table 4:** Results of the Antimicrobial Susceptibility Assay Results for *H. coagulans* AO1167B.

Antimicrobial	M100 Susceptible Breakpoint for *Staphylococcus* *aureus*	*Staphylococcus aureus* ATCC 29213		*H. coagulans* AO1167B	
		Zone Diameter (mm) ^1^	Interpretation	Zone Diameter (mm)	Interpretation
Ampicillin	≥29	41.8	S ^2^	45.3	S
Chloramphenicol	≥19	24.5	S	33.2	S
Clindamycin	NA	29	S	44.1	S
Erythromycin	≥15	28.4	S	41.5	S
Gentamicin	≥18	24.2	S	36.2	S
Kanamycin	NA	24	S	36.2	S
Streptomycin	≥18	17.1	S	25.4	NA ^3^
Tetracycline	≥23	31.9	S	45.8	S
Vancomycin	≥21	18.7	S	36.3	NA

^1^ Zone diameter, mean value of two or more repeat values. ^2^ S, susceptible. ^3^ NA, Kirby–Bauer disk-based breakpoint not available.

**Table 5:** Antimicrobial genes identified by sequencing analysis of the genome of AO1167B.

Gene	Length (bp)	Resistance Mechanism	AMR Gene Function	% Identity of Matching Region
*vanH* gene in *vanO* cluster	990	glycopeptide	glycopeptide resistance gene cluster	31.78
*vanY* gene in *vanM* cluster	795	glycopeptide	glycopeptide resistance gene cluster	35.63
*vanT* gene in *vanG* cluster	1152	glycopeptide	glycopeptide resistance gene cluster	34.38
*vanW* gene in *vanI* cluster	1824	glycopeptide	glycopeptide resistance gene cluster	32.00
*qacG*	744	efflux	small multidrug resistance protein that facilitates the efflux of QACs ^1^	44.4
*qacJ*	744	efflux	small multidrug resistance protein that facilitates the efflux of QACs	46.46
*acrB*	3165	multidrug	resistance-nodulation-cell division (RND) efflux pump	80.41

^1^ QAC = quaternary ammonium compounds.

**Table 6:** Results of Clinical Parameters and Biomarkers of the Study.

Clinical Chemistry and Haematology	Normal Range	*H. coagulans* Cohort (n = 50)		Placebo Cohort (n = 49)		*p* Value			
		Baseline	Day 60	Baseline	Day 60	p1	p2	p3	p4
Creatinine	47.6–113.4 μmol/L	78.23 ± 18.09	75.53 ± 17.22	73.10 ± 21.59	76.167 ± 17.84	NS	NS	0.032	NS
Urea	<8.3 mmol/L	4.27 ± 1.05	4.28 ± 1.01	4.02 ± 1.16	4.47 ± 1.12	NS	NS	NS	0.012
ALAT	<45 U/L	14.98 ± 6.55	13.73 ± 5.78	19.44 ± 9.45	16.26 ± 6.71	0.008	0.047	NS	0.002
ASAT	40 U/L	13.74 ± 4.76	22.83 ± 6.18	18.13 ± 4.78	13.35 ± 5.01	<0.01	<0.01	<0.01	<0.01
GGT	<50 U/L	21.70 ± 16.82	19.64 ± 14.51	22.57± 16.62	24.80 ± 20.21	NS	NS	NS	0.026
Total Protein	60–80 g/L	70.84 ± 4.03	71.07 ± 3.53	70.59 ± 6.22	70.77 ± 4.00	NS	NS	NS	NS
Albumin	35–52 g/L	44.45 ± 3.40	39.92 ± 2.19	44.78 ± 3.38	44.65 ± 2.89	NS	<0.01	<0.01	NS
Glycemia	4.2–6.1 μmol/L	4.70 ± 0.46	4.61 ± 0.60	4.74 ± 0.55	4.69 ± 0.46	NS	NS	NS	NS
Cholesterol	2.81–5.2 mmol/L	4.38 ± 1.15	4.47 ± 0.92	4.95 ± 1.24	4.49 ± 1.11	0.020	NS	NS	<0.01
Triglycerides	0.46–1.8mmol/L	1.131 ± 0.74	1.60 ± 0.52	1.29 ±0.87	1.33 ± 0.69	NS	0.031	0.001	NS
Total bilirubin	<17 mmol/L	8.05 ± 4.47	9.37 ± 3.21	9.32 ± 5.33	7.62 ± 4.49	NS	0.028	0.01	0.002
Direct bilirubin	<5.1 mmol/L	3.02 ± 1.23	2.95 ± 1.13	4.03 ± 4.59	2.95 ± 1.24	NS	NS	NS	0.001
WBC	(4.5–11) × 10^9^/μL	6.37± 2.00	6.31± 2.35	6.26 ± 1.62	6.52 ± 1.96	NS	NS	NS	NS
RBC	(F = 4.2–5.4/M = 4.7–6.1) cels/μL	4.56 ± 0.38	4.55 ± 0.38	4.51 ± 0.40	4.56 ± 0.39	NS	NS	NS	NS
HBG	(F = 12.3–15.3/M = 14.0–17.5) g/dL	132.74 ± 13.65	132.88 ± 13.31	131.53 ± 15.45	132.51 ± 13.71	NS	NS	NS	NS
HTC	(F = 36–45/M = 42–50) %	0.42 ± 0.03	0.42 ± 0.03	0.40 ± 0.04	0.42 ± 0.03	NS	NS	NS	0.001
MVC	80–96.1%	92.57± 5.24	91.49 ± 5.31	90.79 ± 6.15	92.30 ± 5.53	NS	NS	0.003	<0.01
MCH	33.4–35.5 g/dL	29.11 ± 2.05	29.24 ± 1.96	29.15 ± 2.36	29.02 ± 2.13	0.046	0.013	<0.01	NS
PLT	(172–450) × 10^3^/mL	257.70 ± 70.08	267.06 ± 66.51	261.88 ± 52.73	259.67 ± 62.52	NS	NS	NS	NS
RDWCV	(11–14) %	1.05 ± 1.22	12.96 ± 1.08	12.82 ± 1.90	13.12 ± 1.22	NS	NS	0.017	0.014
MPV	(F: 12–16/M: 14–17.4) g/dL	10.45 ± 0.90	10.66 ± 0.88	10.34 ± 0.90	10.35 ± 0.80	NS	NS	0.040	NS
Neutrophil	1.42–6.34 × 10^9^/L	3.60± 1.55	3.65 ± 1.90	3.36 ± 1.16	3.66 ± 1.56	NS	NS	NS	NS
lymphocytes	0.71–4.53 × 10^9^/L	2.02 ± 0.64	1.91 ± 0.63	2.12 ± 0.63	2.10 ± 0.66	NS	NS	NS	NS
Monocytes	0.14–0.72 × 10^9^/L	0.52 ± 0.17	0.55 ± 0.23	0.52 ± 0.13	0.52 ± 0.16	NS	NS	NS	NS
Eosinophils	0–0.54 × 10^9^/L	0.19 ± 0.16	0.19 ± 0.19	0.20 ± 0.17	0.19 ± 0.13	NS	NS	NS	NS
Basophils	0–0.18 × 10^9^/L	0.02 ± 0.01	0.02 ± 0.01	0.02 ± 0.01	0.02 ± 0.01	NS	NS	NS	NS
Bioimpedance Variables		Baseline	Day 60	Baseline	Day 60	p1	p2	p3	p4
Weight		68.49 ± 12.09	66.45 ± 12.60	66.64 ± 13.72	66.43 ± 13.57	NS			
BMI		24.07 ± 3.65	23.13 ± 3.99	23.63 ± 4.17	23.57 ± 4.15	NS			

p1: Analysis of independent samples *H coagulans* group vs. Placebo at baseline. p2: Analysis of independent samples *H coagulans* group vs. Placebo at day 60. p3: Analysis of related samples in *H. coagulans* group baseline day vs. day 60. p4: Analysis of related samples in Placebo baseline day vs. day 60.NS: Non Significative ALAT = alanine transaminase; ASAT = aspartate transaminase; GGT = gamma glutamyl transferase; WBC = White blood cell count; RBC = red blood cell count, HBG = hemoglobin, HTC = hematocrit; MCV = mean corpuscular volume; MCHC = mean corpuscular hemoglobin concentration; PLT = platelet, MPV = mean platelet volume, RDWCV = Red Cell distribution with (Variation in the size of red blood cells); F = female, M = male. BMI = Body Mass Index All values expressed as mean ± SD. The student’s *t*-test for comparing means of independent samples.

**Table 7:** Summary of all adverse effects by intensity.

Adverse Event	*H. coagulans* Cohort (n = 50)			Placebo Cohort (n = 49)		
	Mild	Moderate	Severe	Mild	Moderate	Severe
Abdominal/GI discomfort	2 (4.0)			0	0	0
acne rosacea	0	0	0	0	0	0
Anxiety Depression	0	0	0	0	0	0
Joint pain	0	0	0	0	0	0
Bronchitis	0	0	0	0	0	0
Excision of birthmarks	0	0	0	0	0	0
Bruises after a fall	0	0	0	0	0	0
Crotid stenosis	0	0	0	0	0	0
Cataract Surgery	0	0	0	0	0	0
Chondrocalcinosis	0	0	0	0	0	0
Colonoscopy and fibroscopy	0	0	0	0	0	0
Cystitis	0	0	0	0	0	0
Dental pain	0	0	0	0	0	0
Diarrhoea	3 (6.0)	0	0	2 (2.0)	0	0
Dizziness and nausea	0	0	0	0	0	0
Oedema	0	0	0	0	1 (1.0)	0
Gases	5 (10.0)	0	0	0	0	0
General aches	0	0	0	0	0	0
Genital herpes	0	0	0	0	0	0
Headache	1 (2.0)	0	0	0	0	0
Haemorrhoids	0	0	0	0	0	0
Infection	0	0	0	0	0	0
Inflamed prostate	0	0	0	0	0	0
Migraine	0	0	0	0	0	0
Mouth ulcer	0	0	0	0	0	0
Muscle discomfort	0	0	0	0	0	0
Nasal obstruction	0	0	0	0	0	0
Orthopaedic pain	0	0	0	0	0	0
Pain following capsule consumption	0	0	0	0	0	0
Palpitations	0	0	0	0	0	0
Radio-infiltration (shoulder)	0	0	0	0	0	0
Rhinitis	0	0	0	0	0	0
Sore throat	0	0	0	0	0	0
Tracheitis	0	0	0	0	0	0
Trouble sleeping (insomnia)	0	0	0	0	0	0
Vaginal dryness	0	0	0	0	0	0
Vagal seizures during or after taking a blood sample.	0	0	0	0	0	0
vitamin D deficiency	0	0	0	0	0	0
Others (constipation)	2 (4.0)	0	0	0	0	0

**Table 8:** Summary of SF-36 Results Across Study Groups.

Question	Associated Answer	Placebo (n = 49)	Cases (n = 50)	Kappa Test/*p* Value	
		Before/After	Before/After	Placebo	Cases
1-general, would you say your health is:	Excellent	5/6	4/8	0.94 *p* = 0.000	0.78 *p* = 0.000
	Very Good	21/21	32/28		
	Good	22/21	14/14		
	Fair	2/2	0/0		
6-During the past 4 weeks, to what extent has your physical health or emotional problems interfered with your normal social activities with family, friends, neighbors, or groups?	Not at all	14/36	39/40	0.95 *p* = 0.000	0.82 *p* = 0.000
	Slightly	14/11	11/10		
	Moderately	18/1	0/0		
	Quite a bit	1/0	0/0		
	Extremely	1/0	0/0		
7-How much bodily pain have you had during the past 4 weeks?	None	14/18	19/22	0.82 *p* = 0.000	0.77 *p* = 0.000
	Very mild	14/14	15/16		
	mild	18/14	13/9		
	moderate	1/1	3/3		
	severe	1/1	0/0		
11a-I seem to get sick a little easier than other people	Definitely true	0/0	0/0	0.96 *p* = 0.000	0.92 *p* = 0.000
	Mostly true	3/3	0/0		
	Don’t know	2/2	5/4		
	Mostly False	10/9	12/12		
	Definitely False	33/34	32/33		
11b-I am as healthy as anybody I know	Definitely true	19/19	17/17	0.97 *p* = 0.000	0.97 *p* = 0.000
	Mostly true	16/15	25/26		
	Don’t know	6/6	5/4		
	Mostly False	4/4	1/1		
	Definitely False	3/4	1/1		
11c-I expect my health to get worse	Definitely true	1/1	0/0	0.96 *p* = 0.000	0.91 *p* = 0.000
	Mostly true	0/0	0/1		
	Don’t know	19/18	7/6		
	Mostly False	5/5	6/7		
	Definitely False	23/24	36/35		
11d-My health is excellent	Definitely true	9/10	9/8	0.93 *p* = 0.000	0.92 *p* = 0.000
	Mostly true	27/25	31/33		
	Don’t know	6/6	8/7		
	Mostly False	3/3	1/1		
	Definitely False	3/4	0/0		

## Data Availability

The genomic data supporting the findings of this study are available in the NCBI BioProject SAMN43867243.
